# Ethical and equitable considerations when establishing a diagnosis of an inborn error of immunity

**DOI:** 10.70962/jhi.20250068

**Published:** 2025-08-07

**Authors:** Timothy M. Buckey, Jennifer R. Heimall

**Affiliations:** 1 Division of Allergy and Immunology, Children’s Hospital of Philadelphia, Philadelphia, PA, USA; 2 Section of Allergy and Immunology, Division of Pulmonary, Allergy, and Critical Care Medicine, Perelman School of Medicine, University of Pennsylvania, Philadelphia, PA, USA; 3 Department of Medical Ethics and Health Policy, Perelman School of Medicine, University of Pennsylvania, Philadelphia, PA, USA; 4 Department of Pediatrics, Perelman School of Medicine, University of Pennsylvania, Philadelphia, PA, USA

## Abstract

As a heterogeneous group of disorders, inborn errors of immunity have a variety of diagnostic and management approaches. Clinical immunologists regularly encounter ethical and equitable dilemmas when caring for this population. While some of these predicaments are similar to any person with a chronic condition, there are additional unique challenges faced by those with an inborn error of immunity. Advances in genetic testing have improved the ability to provide precise medical care, and simultaneously they have forced clinical immunologists to contemplate the ethical and equitable consequences of this testing. As diagnostic modalities and therapeutics continue to advance, new ethical and equitable dilemmas will arise. An understanding of medical ethics by clinical immunologists is fundamental for providing patient-centered medical care.

## Teaser

This article describes the ethical dilemmas encountered by clinical immunologists in the evaluation of an inborn error of immunity. Potential sources of health disparities are reviewed. This article describes several key ethical principles and articulates how immunologists can navigate ethical dilemmas in their practice.

Inborn errors of immunity (IEIs), or primary immunodeficiencies, are a heterogeneous group of disorders with variable presentations and severity, as well as diagnostic and management approaches. Often, IEIs are discovered in early childhood before an individual can understand the potential consequences of the diagnosis. There are many equitable and ethical dilemmas when establishing an IEI diagnosis. Access to care, time, and financial expenses are potential barriers for any individual with a chronic medical condition. Individuals with IEIs encounter these same challenges while facing additional unique predicaments. This article will first describe the ethical principles pertinent to the immunologic evaluation, then discuss practical dilemmas with testing, and lastly discuss the problem of access to clinical immunologists and genetic counselors. This article will demonstrate familiarity with medical ethics is a necessity for the daily practice of clinical immunologists (1).

When performing an IEI evaluation, clinical immunologists must remember both anticipated and unintended results may have lifelong consequences. Thus, testing modalities should be selected using an ethical framework. In accordance with the principles of beneficence and nonmaleficence, testing should aim to maximize benefit and minimize harms (2, 3). Beneficence is defined as acting to promote the health of the patient, whereas nonmaleficence is refraining from causing injury or harm to the patient (2, 3).

Consent and assent are essential aspects of the evaluation (3). Informed consent entails a patient understands a procedure’s rationale, benefits, risks, and alternatives, and then the patient provides permission for it to occur. With children, caregivers provide consent. Pediatric assent, or provision of affirmation, is sought but not always required, for example, if the child is too young (4). Ideally, the child assents and the parent consents. The distinction between consent and assent is important because parental decisions made early in children’s lives can have lasting implications. If clinical immunologists encounter a scenario in which children and their caregivers have different preferences, the physician should seek to understand both parties’ values and engage in shared decision-making.

Justice is the notion that the provision of healthcare should be equitable and fair (2, 3). Immunologic testing can be time- and resource-intensive and requires interpretation by trained specialists. Additionally, IEIs often have multi-organ manifestations requiring the coordinated and collaborative care of clinicians of multiple specialties. Hence, clinical immunologists routinely encounter dilemmas on the practical application of justice with respect to decisions on the distribution of limited healthcare resources.

Also, trust between patient and physician should be emphasized (3). Physicians who demonstrate an empathetic approach with active listening provide the opportunity for patients to express their values so that these can be incorporated in the evaluation. Patients come from all backgrounds, and medical mistrust exists due to a history of biased and unethical practices (5). Mistrust could present as hesitancy to receive vaccinations, take medications, or undergo genetic testing. As patients with IEIs require lifelong follow-up, trust in healthcare is essential (3). Moreover, once they reach adulthood, these individuals will transition to having the primary responsibility for managing their condition, as opposed to their parents during childhood. Thus, providing education and maintaining trust are central skills for clinical immunologists.

It has been shown that there are disparities in IEI-associated mortality based on race and ethnicity (6). Access, including time, financial, and physician availability, is a potential source of health disparities. The immunologic evaluation often requires multiple forms of testing and visits, resulting in direct and indirect costs to patients. Immunologic testing is expensive, and based on insurance, patients can confront high costs. Missing work, and the resultant lost income, to attend visits is another burden. For rural settings, traveling to see a clinical immunologist is a potential obstacle. While telemedicine has alleviated this burden for many, the inability to perform telehealth visits across state lines is a source of health inequity (7). Moreover, as there is uncertainty regarding the long-term availability of telehealth, this is one example in which clinical immunologists can advocate for access to medical care. Further research into understanding health disparities among individuals with IEIs is needed and could present opportunities for physicians to advocate on behalf of their patients.

There are unique challenges encountered during the evaluation of a potential IEI in comparison with other chronic conditions. Immunologic testing can be expensive and frequently requires specialized, coordinated testing, as well as the need for matched healthy controls, such as with the dihydrorhodamine test in the chronic granulomatous disease evaluation. Unlike routine laboratory tests, such as a complete blood count, a person’s genetic makeup is intrinsic information that does not change over their lifetime. By performing genetic testing during childhood, clinicians and caregivers may reveal information requiring lifelong monitoring and future consequences.

Clinical immunologists regularly encounter dilemmas on the benefits and harms of various tests and their clinical application. Using genetic testing as an example, clinical immunologists face quandaries over if, when, and which types of testing to perform. There are numerous benefits of genetic testing as advances in genetics have provided opportunities to diagnose and provide precise, pathway-based treatments, thus standardizing care. Moreover, genetic testing can allow a diagnosis to be made before physical manifestations or a severe infection develops; for example, by detecting an IEI pathogenic mutation that would benefit from bone marrow transplant (BMT), testing provides the opportunity to deliver a lifesaving procedure before the acquisition of a critical infection. Severe combined immunodeficiency is a fundamental example of this concept. Yet, even with these benefits, clinical immunologists must appropriately utilize genetic testing, counsel that testing does not always identify a diagnosis, and explain forgoing testing is an option.

When a clinical immunologist contemplates ordering a gene panel versus a whole-exome sequencing, there are equitable and ethical implications with both forms of testing. The former may be associated with decreased costs, faster results, and increased access. Yet, the latter provides more extensive information. With more focused testing, it is possible to miss a diagnosis resulting in uncertainty, caregiver anxiety, and delayed treatment. Yet, more extensive testing has the potential to yield unintended results, such as pathogenic mutations or variants of uncertain significance in unanticipated genes. With any testing method, if an IEI diagnosis is made it can have lifelong consequences with potential health implications and requiring clinical monitoring. For example, common variable immunodeficiency (CVID) is associated with increased risks for autoimmunity, bronchiectasis, and malignancy. Therefore, individuals with CVID require monitoring for the development of these conditions. Notably, even if a known pathogenic variant is identified, outcomes and severity of IEIs are variable, and this emphasizes the fact that ethical dilemmas and some uncertainties are intrinsic to the practice of clinical immunology. This concept can be applied to IEIs in which BMT is pursued. While BMT is potentially a lifesaving intervention, it can be complicated by a lack of suitable donors, adverse effects of medications, and potential infections before and after transplant.

Additionally, while there has been research showing adverse psychological effects associated with genetic testing in children are uncommon, these studies have focused on conditions other than IEIs and there is limited longitudinal research (8). Thus, a topic for future research is to assess the long-term psychological impacts of genetic testing for both individuals with IEIs and family members of those with IEIs.

Another area of ethical complexity that clinical immunologists routinely encounter is how to approach previous immunologic evaluations. If testing was performed years ago and was non-diagnostic, it raises the predicament if it is ethical to rely on those results since knowledge of genetics and the immune system is constantly evolving. Yet, asking patients to undergo repeated rounds of testing could further exacerbate health disparities.

Approximately 150,000–200,000 individuals in the United States have an IEI (9). In the United States, there are limited allergy/immunology training programs and there is an expected shortage of clinical immunologists (10). Currently, there are 7,282 physicians certified in allergy/immunology, some of whom may have a primary clinical focus on allergy (11). Moreover, while clinical immunologists ought to be familiar with genetic testing, genetic counselors are skilled at providing guidance on genetic testing, their results, and consequences. Thus, they have an integral role in the diagnosis and management of IEIs. In 2021, there were 5,629 certified genetic counselors (12). Despite the many individuals in the United States with an IEI, there is a clear shortage of both clinical immunologists and genetic counselors to care for these patients. This is further exacerbated by the fact that these clinicians can be concentrated in cities and academic institutions and therefore are not equally accessible to people across the country. Thus, there are both current concerns about access to pediatric immunologists and an impending predicament of access to adult immunologists when children with IEIs transition to adulthood.

Due to ongoing advances in technology, new ethical dilemmas with practical implications for patients and clinical immunologists will arise. Also, as different genetic and immunologic testing modalities become widely available there will be additional questions of access, affordability, and health literacy underpinning decisions on which tests to perform. Clinical immunologists and genetic counselors will have to participate in shared decision-making with patients on the use of these various modalities. Another forthcoming predicament is access to reproductive counseling for patients with an IEI if they consider having children when they reach adulthood. Clinical immunologists will continue to encounter increasingly complex ethical predicaments as to how to balance accurately diagnosing IEIs while respecting fundamental ethical principles (2).

The heterogeneity of IEIs creates ethical and equitable dilemmas as there is not a single correct diagnostic or management approach. While potentially lifesaving, testing and subsequent interventions nevertheless create ethically complex scenarios that clinical immunologists routinely encounter and may have lifelong consequences for patients. Thus, medical ethics is increasingly under the purview of clinical immunologists and should be a focus of clinician education and training.

## Data Availability

No new data were generated or analyzed in support of this study.
